# COVID-19 and Aging-Related Genome (Chromosome) Instability in the Brain: Another Possible Time-Bomb of SARS-CoV-2 Infection

**DOI:** 10.3389/fnagi.2022.786264

**Published:** 2022-03-03

**Authors:** Ivan Y. Iourov, Svetlana G. Vorsanova

**Affiliations:** ^1^Yurov's Laboratory of Molecular Genetics and Cytogenomics of the Brain, Mental Health Research Center, Moscow, Russia; ^2^Laboratory of Molecular Cytogenetics of Neuropsychiatric Diseases, Veltischev Research and Clinical Institute for Pediatrics of the Pirogov Russian National Research Medical University, Moscow, Russia; ^3^Department of Medical Biological Disciplines, Belgorod State University, Belgorod, Russia

**Keywords:** aging, brain, chromosome instability, COVID-19, neurodegeneration, genome instability, SARS-CoV-2

It is hard to estimate the profound impact of COVID-19 (SARS-CoV-2 infection) on our life. SARS-CoV-2 infection is the cause of a pandemic and is associated with a severe disease threatening life during and after the manifestation (Tu et al., 2020; Wu et al., 2020; Hu et al., 2021). It is repeatedly noted that the central nervous system is seriously affected by COVID-19 infection. However, the intrinsic effects of COVID-19 on the human brain remain a matter of future research (Pennisi et al., 2020). Pathological brain aging and natural brain deterioration are associated with accumulation and propagation of genome (chromosome) instability (Yurov et al., 2010, 2019; Andriani et al., 2017; Zhang and Vijg, 2018; Iourov et al., 2021). Since genome (chromosome) instability may result from viral infections (Heng, 2019), SARS-CoV-2 interactions with cells of the central nervous system are able to increase the risk for early manifestations of aging-related brain disorders and/or premature brain deterioration mediated by genome and chromosome instability. Recently, a number of studies dedicated to host-coronavirus protein interaction networks have highlighted numerous molecular and cellular processes, which are likely to be altered by SARS-CoV-2 (Gordon et al., 2020a; Lee et al., 2021; Schmidt et al., 2021; Terracciano et al., 2021). Here, we have addressed data on SARS-CoV-2–host protein–protein interactomes for assessing potential COVID-19 effects on aging-related genome (chromosome) instability in the brain.

Data on SARS-CoV-2–host protein–protein interactomes or networks were taken from following articles: Díaz (2020); Gordon et al. (2020a,b); Guzzi et al. (2020); Perrin-Cocon et al. (2020); Schmidt et al. (2021), and Terracciano et al. (2021). Candidate pathways (networks) were grouped according to the association with processes involved in genome stability maintenance, cell cycle regulation, chromatin regulation, DNA metabolism, and cell death. These clusters of pathways are generally associated with brain-specific chromosome/genome instability.

Looking through the SARS-CoV-2–host interactomes, one may find a wide spectrum of different pathways affected by the coronavirus. For more details, see Díaz (2020); Gordon et al. (2020b), and Terracciano et al. (2021). However, each article reported on a small but significant proportion of pathways implicated in genome stability maintenance, DNA regulation, and chromatin organization. Taking into account previous evaluations of processes involved in brain-specific chromosome/genome instability (Jeppesen et al., 2011; Yurov et al., 2011; Bajic et al., 2015; Caneus et al., 2018; Martínez-Cué and Rueda, 2020; Heng et al., 2021), following candidate pathways (pathway clusters) were selected: cell cycle, cell death, centrosome, chromatin organization, DNA damage response, DNA regulation, DNA replication, DNA repair, ER stress, and nucleotide metabolism. The rates of chromosome and genome instability may increase with age being highly dependent on environmental factors (Iourov et al., 2020; Vorsanova et al., 2020). A viral infection may be such a factor (Heng et al., 2021). Therefore, according to interactomic data, SARS-CoV-2 interactions with proteins involved in the aforementioned pathways are able to initiate/stimulate genome and chromosome instability in neuronal cells. Figure 1 schematically depicts possible effects of SARS-CoV-2 infection on the brain in the context of aging-related genome (chromosome) instability.

**Figure 1:**
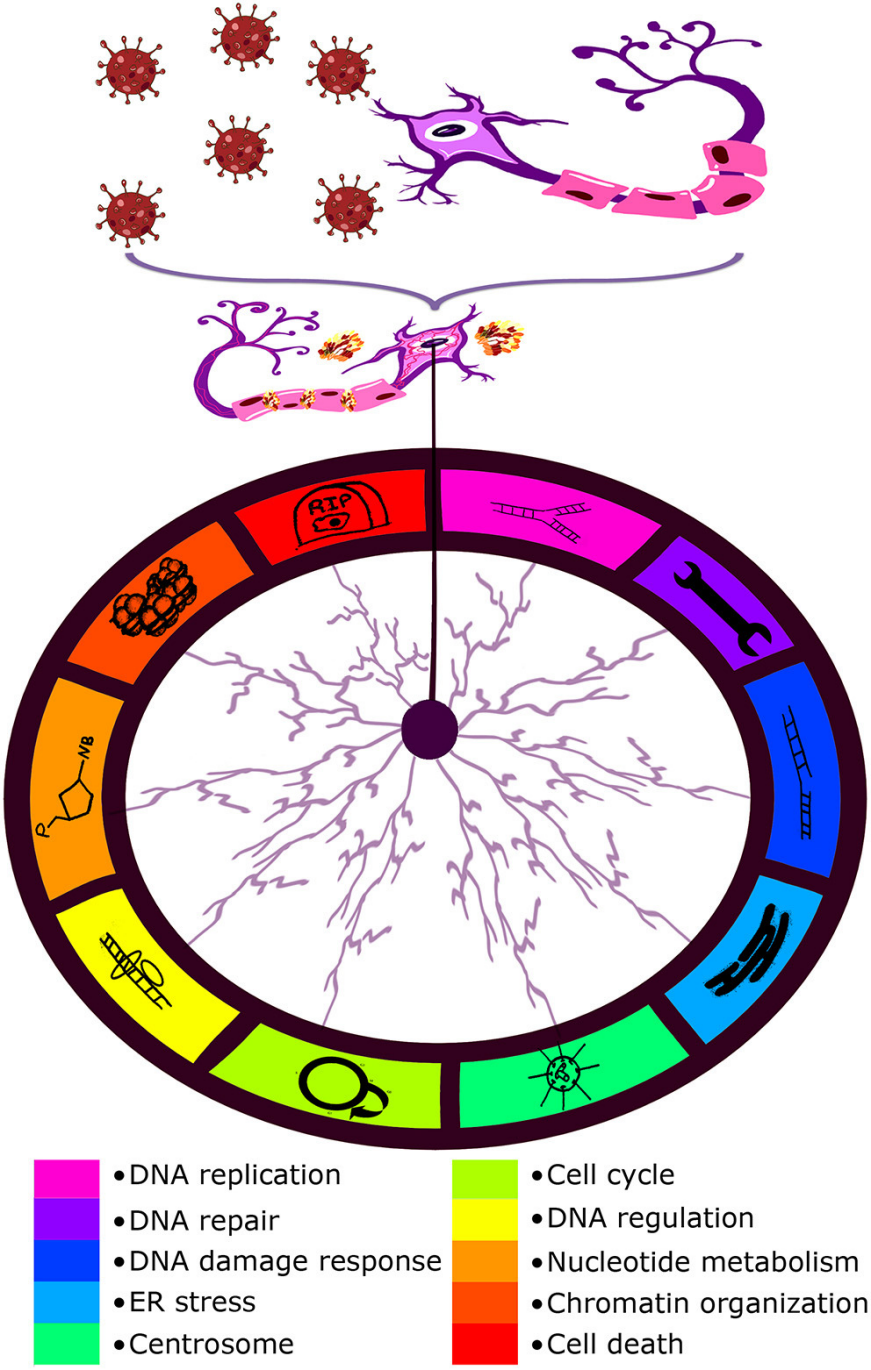
Schematic representation of the possible effect of SARS-CoV-2 infection (COVID-19) on the brain in the context of aging-related genome (chromosome) instability. Interactome analysis of SARS-CoV-2 infection has highlighted a number of pathways to be potentially altered by the virus which are listed at the bottom of the figure.

Brain-specific genomic variations [including aneuploidy (loss/gain of whole chromosomes) and single gene mutations] are associated with a wide spectrum of late-onset brain diseases (Yurov et al., 2010; Rohrback et al., 2018; Iourov et al., 2021). More importantly, chromosome instability mediates neurodegeneration (Iourov et al., 2009; Rohrback et al., 2018; Yurov et al., 2019). Several molecular pathways have been associated with chromosome instability in the neurodegenerating brain including neuronal cell cycle errors, chromosome missegregation, and cellular senescence (Bajic et al., 2015; Caneus et al., 2018; Martínez-Cué and Rueda, 2020). These processes are intimately linked to aging at molecular, cellular, and tissular levels. For instance, premature aging is associated with increased rates of chromosome and genome instability. Natural aging is associated with accumulation and propagation of somatic genome variations (e.g., aneuploidy) and genome instability. Alterations to genome stability maintenance may cause aging-related brain diseases or early manifestations of late-onset neurodegenerative diseases (Yurov et al., 2010; Andriani et al., 2017; Zhang and Vijg, 2018; Iourov et al., 2021). Additionally, DNA regulation and chromatin organization are able to affect genome stability by altering the expression of genes implicated in the pathways demonstrated in Figure 1. Since SARS-CoV-2 interactions with proteins involved in genome stability maintenance pathways are able to contribute to chromosome/genome instability propagation, the coronavirus infection has the potential to cause neurobehavioral abnormalities, neurodegeneration (e.g., Alzheimer's disease) and premature brain deterioration.

SARS-CoV-2 infection possesses an appreciable effect on the organism (Tu et al., 2020; Wu et al., 2020; Hu et al., 2021). Alterations to the central nervous system are observed in individuals with COVID-19 (Pennisi et al., 2020). Here, we express our opinion that SARS-CoV-2 may increase the risk for neurobehavioral alterations and neurodegeneration mediated by aging-related genome (chromosome) instability. Thus, SARS-CoV-2 is a potential risk factor for premature brain deterioration, Alzheimer's disease and other late-onset neurodegenerative diseases. The opinion is supported by addressing SARS-CoV-2-host interactomes. It is to note that chromosome instability mediating complex diseases and aging is not specific for brain diseases (Yurov et al., 2010; Iourov et al., 2019, 2020; Vorsanova et al., 2020). In other words, similar processes may occur in any tissue of an individual with SARS-CoV-2 infection. Additionally, other viruses are able to produce chromosome instability (Heng, 2019). Thus, one should be aware of the complications caused by chromosome and genome instability (e.g., cancer or tissue degeneration) in individuals affected by COVID-19 infection. Moreover, these studies seem to be especially important for those who went through a cytokine storm, since the latter may trigger tissue degeneration. Accordingly, molecular cytogenetic monitoring of chromosome and genome instability is warranted in individuals with SARS-CoV-2 infection to prevent genome instability-mediated and aging-dependent pathologies on time.

## Author Contributions

II wrote the manuscript. Both authors conceived the idea and made theoretical contributions.

## Funding

Authors were partially supported by RFBR and CITMA according to the Research Project No. 18-515-34005. The work was partially supported by the Government Assignment of the Russian Ministry of Science and Higher Education, Assignment No. AAAA-A19–119040490101-6 (Mental Health Research Center) and by the Government Assignment of the Russian Ministry of Health, Assignment No. 121031000238-1 (Veltischev Research and Clinical Institute for Pediatrics).

## Conflict of Interest

The authors declare that the research was conducted in the absence of any commercial or financial relationships that could be construed as a potential conflict of interest.

## Publisher's Note

All claims expressed in this article are solely those of the authors and do not necessarily represent those of their affiliated organizations, or those of the publisher, the editors and the reviewers. Any product that may be evaluated in this article, or claim that may be made by its manufacturer, is not guaranteed or endorsed by the publisher.
